# Development and validation of a machine learning-based predictive model for chemotherapy-induced myelosuppression in colorectal cancer patients

**DOI:** 10.3389/fmed.2026.1778951

**Published:** 2026-03-25

**Authors:** Xue Song, Shuwen Li, Feng Li, Yulin Chai, Jianjun Wang, Juanjuan Zu

**Affiliations:** 1 School of Nursing, The First Affiliated Hospital of Anhui Medical University, Hefei, China; 2 Department of Oncology, The First Affiliated Hospital of Anhui Medical University, Hefei, China; 3 The First Department of General Surgery, Changfeng County People's Hospital, Hefei, China; 4 Department of Oncology, Feidong County People's Hospital, Hefei, China

**Keywords:** chemotherapy-induced myelosuppression, colorectal cancer, machine learning, predictive model, random forest, SHAP analysis

## Abstract

**Objective:**

This study aimed to develop and validate a machine learning–based predictive model for individualized assessment and management of chemotherapy-induced myelosuppression (CIM) in patients with colorectal cancer (CRC).

**Methods:**

A total of 450 patients with CRC undergoing chemotherapy were retrospectively enrolled in the training cohort, and an additional 150 patients were included for external validation. Feature selection was performed using the Least Absolute Shrinkage and Selection Operator (LASSO) regression. Three machine learning algorithms [Logistic Regression (LR), Random Forest (RF), and Extreme Gradient Boosting (XGBoost)] were applied to construct predictive models. Model performance was assessed using multiple metrics, including accuracy, area under the receiver operating characteristic curve (AUC), F1 score, sensitivity, and specificity. The SHapley Additive exPlanations (SHAP) method was employed to rank and interpret the importance of predictive features.

**Results:**

In the training cohort, 52.4% of patients developed CIM. The feature selection process identified 19 significant variables that were incorporated into the predictive models. Both LR and RF demonstrated optimal performance, with an AUC of 0.83 and an accuracy of 0.76 in the training set. In the test set, RF continued to outperform other models, achieving an AUC of 0.77 and an accuracy of 0.71. External validation confirmed the robustness of the RF model, which achieved an AUC of 0.93 (95% CI: 0.89–0.97), an accuracy of 0.89, a sensitivity of 0.86, and a specificity of 0.92. SHAP analysis revealed that the most important predictors included hematological parameters, nutritional risk score (NRS2002), and a history of radiotherapy.

**Conclusion:**

The RF-based machine learning model demonstrated high accuracy and strong external validation capability for predicting the risk of CIM in CRC patients.

## 1 Introduction

CRC represents a significant global health burden, with over 1.9 million new cases and approximately 935,000 deaths in 2020 (1). It ranks third in cancer incidence and second in cancer-related mortality worldwide, and second in incidence and fifth in mortality in China (2, 3). Chemotherapy is a primary treatment for CRC, especially for advanced or high-risk patients, with regimens such as FOLFOX, CAPOX, and FOLFIRI often combined with targeted therapies like bevacizumab or cetuximab (4). However, chemotherapy frequently causes chemotherapy-induced myelosuppression (CIM), a severe side effect characterized by neutropenia, anemia, and thrombocytopenia (5). CIM increases the risk of infections, bleeding, and treatment delays (6, 7), while also contributing to fatigue, anxiety (8), and reduced quality of life (9). It also leads to longer hospital stays, higher healthcare costs, and greater burdens on patients and their families. Therefore, early identification and individualized risk prediction of CIM are critical for preventing adverse outcomes and enhancing supportive care interventions.

Although several predictive models for CIM have been developed for cancer patients undergoing chemotherapy, most studies have primarily focused on cancers such as lung (10), breast (11), or esophageal cancer (12), with relatively few addressing CRC patients (13–15). Furthermore, several critical issues remain unresolved in the existing predictive models. First, some models are limited to predicting specific hematological toxicities, most commonly neutropenia or thrombocytopenia (16, 17), without encompassing the full spectrum of CIM, thus restricting their clinical applicability. Second, most models are built using data from single center or internal datasets, lacking external validation (18), which severely limits their generalizability across different regions and institutions. Additionally, some studies focus on basic hematological parameters and chemotherapy regimens, neglecting other influential factors such as nutritional status (6). Therefore, it is essential to develop more comprehensive and innovative predictive models that integrate multiple influencing factors, to improve both the accuracy and clinical applicability of CIM prediction.

Machine learning (ML) has gained traction in clinical care, providing novel approaches for risk management in CRC patients undergoing chemotherapy (19–21). Unlike traditional models, ML algorithms can efficiently process large, complex datasets, capturing nonlinear relationships and intricate variable interactions (22). By integrating multidimensional data—such as demographics, laboratory values, and treatment parameters—ML models offer more accurate, individualized predictions, improving predictive accuracy, generalizability, and sensitivity for early detection of toxic events. Among ML algorithms, LR is computationally efficient, offering interpretable coefficients for binary classification problems (23), such as predicting CIM. RF resists overfitting by aggregating multiple decision trees, providing intuitive feature importance rankings (24). XGB excels with structured data, incorporating a unique regularization mechanism to balance model complexity and prevent overfitting, making it suitable for large-scale, big data applications (25).

Although more advanced models such as neural networks may offer strong predictive performance, their lack of interpretability limits their clinical utility in settings where causal inference is essential (26). Moreover, emerging ML algorithms often require specialized hardware, posing implementation challenges in routine clinical practice (27). Therefore, this study selected LR, RF, and XGB as the three primary algorithms to construct and validate a predictive model for CIM in CRC patients. To improve generalizability, we externally validated the model in two county-level hospitals, ensuring its applicability in primary care settings.

## 2 Methods

### 2.1 Data sources

The dataset for model training and test was derived from The First Affiliated Hospital of Anhui Medical University, a tertiary A-level hospital in China. Electronic medical records of outpatient and inpatient CRC patients treated with chemotherapy between January 2020 and December 2023 were retrospectively collected to form the training and test sets. To assess the model’s generalizability, additional data were obtained from Feidong County People’s Hospital and Changfeng County People’s Hospital, which provided clinical data of CRC patients treated with chemotherapy between January 2024 and December 2024, serving as the external validation cohort. The study protocol was approved by the Ethics Committee of The First Affiliated Hospital of Anhui Medical University (approval number: [PJ-2024-12-79]), which served as the central Institutional Review Board (IRB). Data collection from the external validation centers (Feidong County People’s Hospital and Changfeng County People’s Hospital) was conducted under administrative authorizations and strict data-sharing agreements supervised by the central IRB, ensuring adherence to the Declaration of Helsinki and patient privacy protection. Due to the retrospective nature of the study using anonymized data, the requirement for informed consent was waived. The reporting of this study adheres to the TRIPOD statement.

### 2.2 Study population

The inclusion criteria for this study were patients aged 18 years or older, with a histopathologically confirmed diagnosis of colorectal cancer, who had received at least one full cycle of systemic chemotherapy during the study period. Additionally, patients needed to have complete hematological laboratory data available, both within 3 days before the initiation of chemotherapy and within 7 days post-chemotherapy, including baseline and follow-up results. Exclusion criteria consisted of patients with incomplete chemotherapy treatment records, those who received only radiotherapy or targeted therapy without systemic chemotherapy during the study period, patients with concurrent active hematologic malignancies or known bone marrow disorders, and those with missing key covariates, such as peripheral blood counts or medication records and patients who received primary prophylactic treatment with G-CSF or other hematopoietic growth factors.

### 2.3 Data collection

This study identified key variables through a review of existing literature and consultation with clinical experts, categorizing the data into four domains: patient demographic information, tumor-related variables, chemotherapy-related variables, and laboratory indicators. Patient demographic variables included sex, age, body mass index (BMI), Nutritional Risk Screening score (NRS2002), education level, history of alcohol consumption, and the presence of comorbidities such as hypertension, diabetes mellitus, and coronary artery disease. Tumor-related variables encompassed cancer history, prior surgical history and type of surgery, clinical tumor stage, presence of hepatic, osseous, lymphatic, and pulmonary metastases, and maximum tumor diameter. Chemotherapy-related variables included the chemotherapy regimen administered, history of radiotherapy, prior episodes of myelosuppression, and the number of chemotherapy cycles received. Laboratory variables comprised key hematologic and biochemical indices, including white blood cell count (WBC), hemoglobin (Hb), platelet count (PLT), neutrophil count (NEUT), monocyte count (MONO), lymphocyte count (Lym), albumin (ALB), globulin (GLB), blood urea nitrogen (BUN), carcinoembryonic antigen (CEA), carbohydrate antigen 19–9 (CA199), alpha-fetoprotein (AFP), carbohydrate antigen 125 (CA125), total bilirubin (Tbil), serum creatinine (Scr), uric acid (UA), and estimated glomerular filtration rate (GFR).

The primary outcome, CIM, was classified according to WHO grading criteria into four severity grades. Specifically, leukopenia was defined as WBC < 4.0 × 10^9^/L, anemia as Hb < 110 g/L, and thrombocytopenia as PLT < 100 × 10^9^/L. To ensure data harmonization and reproducibility, these standardized cutoffs were strictly applied across the training, internal testing, and external validation cohorts. If any hematologic indicator met Grade I or higher, the patient was classified as having developed CIM. Laboratory variables used as input features for the predictive model were strictly limited to baseline values collected within 3 days prior to the chemotherapy cycle (Day −1). Post-treatment laboratory data (collected on Day +7) were used solely to assess the primary outcome (occurrence of CIM) and were not included as predictors, and missing or erroneous data were flagged and manually verified.

### 2.4 Statistical analysis

The patient was defined as the unit of analysis. For each participant, only the baseline clinical and laboratory data prior to the first chemotherapy cycle were used as predictors. A CIM event was recorded if the patient met the WHO criteria at any time during their treatment, ensuring each individual contributed a single, independent data point.

Data preprocessing was performed using R (version 4.4.2), including data cleaning, transformation, and normalization. Variables with >20% missingness (e.g., tumor size) were excluded. For other variables with <20% missingness, Multiple Imputation by Chained Equations (MICE, *m* = 5) was applied. Crucially, the imputation model was developed using only the training cohort and then applied to the testing and external validation cohorts to prevent data leakage.

Descriptive statistical analyses were conducted using SPSS (version 25.0), with continuous variables expressed as mean ± standard deviation and compared using *t*-tests, non-normally distributed variables reported as medians with interquartile ranges, and categorical variables summarized as frequencies and percentages with Chi-square tests. LASSO regression in R was used for feature selection to identify key factors associated with CIM, providing the basis for model development.

### 2.5 Feature selection

All sample data were randomly split into a training set and a validation set in a 7:3 ratio. To identify the most predictive features while minimizing complexity, LASSO regression was applied to the training set using the glmnet package in R. A 10-fold cross-validation procedure was used to select the optimal regularization parameter (*λ*), aiming to minimize binomial deviance. The final set of predictive features was determined based on the λ that yielded the best model fit.

### 2.6 Machine learning models and evaluation

Three well-established machine learning algorithms—LR, RF, and XGB, were employed to predict the risk of CIM in patients with CRC. Model training was conducted using the optimal feature subset derived from the prior feature selection process. Hyperparameters for the machine learning models were optimized via grid search with 10-fold cross-validation to ensure robustness and prevent overfitting. No class balancing techniques were required due to the naturally balanced dataset. Finally, the optimal probability thresholds for classification were determined using Youden’s Index. Model performance was comprehensively evaluated using multiple metrics, including accuracy, AUC, F1 score, sensitivity, and specificity. Ultimately, the predictive power and calibration performance of each model were compared across both the training and test sets. The model with the best overall performance was selected for subsequent model interpretation and external validation.

### 2.7 Model interpretation (SHAP interpretability analysis)

To enhance model transparency and clinical interpretability, SHAP values were used to quantify the impact of each predictive factor on CIM risk prediction. Visualization, performed using the R packages “shapviz” and “ggplot2,” included summary plots and beeswarm diagrams, illustrating feature importance and their directional influence on model outputs. SHAP analysis clarifies each variable’s contribution to the prediction, adding interpretability to the complex machine learning models. This improves clinician trust in the model and provides valuable insights for developing personalized treatment strategies, making SHAP interpretation a practical tool in oncology patient management.

## 3 Results

### 3.1 Baseline characteristics

A total of 450 CRC patients who received chemotherapy were retrospectively analyzed. The mean age of the entire cohort was 61.90 ± 11.35 years. Patients were divided into two groups based on the occurrence of CIM: a myelosuppression group and a non-myelosuppression group. A total of 236 patients (52.4%) developed myelosuppression, with a mean age of 62.62 ± 11.07 years. Among them, 153 were male (64.83%) and 83 were female (35.17%). In contrast, 214 patients (47.55%) did not experience myelosuppression, with a mean age of 61.10 ± 11.62 years; 139 were male (64.95%) and 75 were female (35.05%).

Comparative analysis of baseline characteristics between the two groups revealed statistically significant differences in the following variables: BMI, bone metastases, history of radiotherapy, prior myelosuppression, NRS2002, WBC, Hb, PLT, NEUT, Lym, ALB, and UA (*p* < 0.05). No significant differences were found for the remaining variables (*p* > 0.05) (Table 1).

**Table 1:** Comparison of baseline data between the two groups in training and testing set [*n* (%), *M* (P25, P75), χ^2^ ± s].

Characteristic	Overall *N* = 450^1^	Non-myelosuppression *N* = 214^1^	Myelosuppression *N* = 236^1^	*p*-value^2^
Gender				>0.9
Male	292 (65%)	139 (65%)	153 (65%)	
Female	158 (35%)	75 (35%)	83 (35%)	
Age	61.9 (11.3)	61.1 (11.6)	62.6 (11.1)	0.2
BMI	23.0 (3.4)	23.4 (3.5)	22.6 (3.3)	0.029
Educational level				0.2
Primary school or less	179 (40%)	76 (36%)	103 (44%)	
Junior high school	137 (30%)	66 (31%)	71 (30%)	
Senior high school	57 (13%)	32 (15%)	25 (11%)	
Junior college or above	77 (17%)	40 (19%)	37 (16%)	
Use of alcohol	59 (13%)	31 (14%)	28 (12%)	0.4
Use of tobacco	57 (13%)	30 (14%)	27 (11%)	0.4
Hypertension	136 (30%)	65 (30%)	71 (30%)	>0.9
Diabetes	57 (13%)	28 (13%)	29 (12%)	0.8
CHD	35 (7.8%)	20 (9.3%)	15 (6.4%)	0.2
With other tumors	26 (5.8%)	13 (6.1%)	13 (5.5%)	0.8
Prior operations	208 (46%)	106 (50%)	102 (43%)	0.2
Surgery method				0.2
No surgery	153 (34%)	64 (30%)	89 (38%)	
Radical operation	207 (46%)	102 (48%)	105 (44%)	
Palliative operation	89 (20%)	47 (22%)	42 (18%)	
Stage of colorectal cancer				0.6
II stage	31 (6.9%)	17 (7.9%)	14 (5.9%)	
III stage	113 (25%)	50 (23%)	63 (27%)	
IV stage	306 (68%)	147 (69%)	159 (67%)	
Hepatic metastases	195 (43%)	85 (40%)	110 (47%)	0.14
Osseous metastasis	24 (5.3%)	5 (2.3%)	19 (8.1%)	0.007
Lymph node metastasis	362 (80%)	176 (82%)	186 (79%)	0.4
Metastatic tumor of lung	93 (21%)	46 (21%)	47 (20%)	0.7
Treatment regimen				0.3
Chemotherapy	255 (57%)	117 (55%)	138 (58%)	
Chemotherapy targeted therapy	169 (38%)	87 (41%)	82 (35%)	
Chemotherapy Immunotherapy	26 (5.8%)	10 (4.7%)	16 (6.8%)	
History of radiotherapy	70 (16%)	23 (11%)	47 (20%)	0.007
History of myelosuppression	118 (26%)	29 (14%)	89 (38%)	<0.001
Chemotherapy cycle				0.2
Less than 6 M	202 (45%)	90 (42%)	112 (47%)	
More than 6 M	248 (55%)	124 (58%)	124 (53%)	
NRS2002				<0.001
Less than 3	243 (54%)	148 (69%)	95 (40%)	
More than 3	207 (46%)	66 (31%)	141 (60%)	
WBC low	123 (27%)	21 (9.8%)	102 (43%)	<0.001
HB low	89 (20%)	11 (5.1%)	78 (33%)	<0.001
PLT low	43 (9.6%)	3 (1.4%)	40 (17%)	<0.001
NEUT low	98 (22%)	19 (8.9%)	79 (33%)	<0.001
Mono high	51 (11%)	24 (11%)	27 (11%)	>0.9
Lym low	44 (9.8%)	9 (4.2%)	35 (15%)	<0.001
ALB low	44 (9.8%)	7 (3.3%)	37 (16%)	<0.001
GLB high	115 (26%)	59 (28%)	56 (24%)	0.4
BUN low	28 (6.2%)	15 (7.0%)	13 (5.5%)	0.5
CEA high	166 (37%)	79 (37%)	87 (37%)	>0.9
CA199 high	122 (27%)	53 (25%)	69 (29%)	0.3
AFP high	90 (20%)	42 (20%)	48 (20%)	0.9
CA125 high	42 (9.3%)	18 (8.4%)	24 (10%)	0.5
TBIL	12.8 (10.0, 16.6)	12.9 (10.3, 16.3)	12.8 (9.5, 16.9)	0.6
Scr	66.0 (52.7, 76.2)	66.3 (54.8, 76.4)	65.9 (51.0, 75.8)	0.6
UA	300.0 (246.0, 357.0)	311.0 (259.6, 367.8)	292.6 (236.5, 338.1)	0.004
GFR	96.9 (16.5)	98.2 (15.6)	95.8 (17.3)	0.3

1 Mean (SD), Median (Q1, Q3); *n* (%).

2 Pearson’s Chi-squared test; Wilcoxon rank sum test.

Data are presented as *n* (%) for categorical variables and mean ± standard deviation (SD) or median (interquartile range [IQR]) for continuous variables, as appropriate. The *p*-values are derived using Pearson’s Chi-squared test for categorical variables and Wilcoxon rank sum test for continuous variables. BMI, body mass index; CHD, coronary heart disease; NRS2002, Nutritional Risk Screening 2002; WBC, white blood cell; Hb, hemoglobin; PLT, platelet; NEUT, neutrophil; MONO, monocyte; Lym, lymphocyte; ALB, albumin; GLB, globulin; BUN, blood urea nitrogen; CEA, carcinoembryonic antigen; CA199, carbohydrate antigen 19–9; AFP, alpha-fetoprotein; CA125, carbohydrate antigen 125; TBIL, total bilirubin; Scr, serum creatinine; UA, uric acid; GFR, glomerular filtration rate.

### 3.2 Feature selection based on LASSO regression

**Figure 1:**
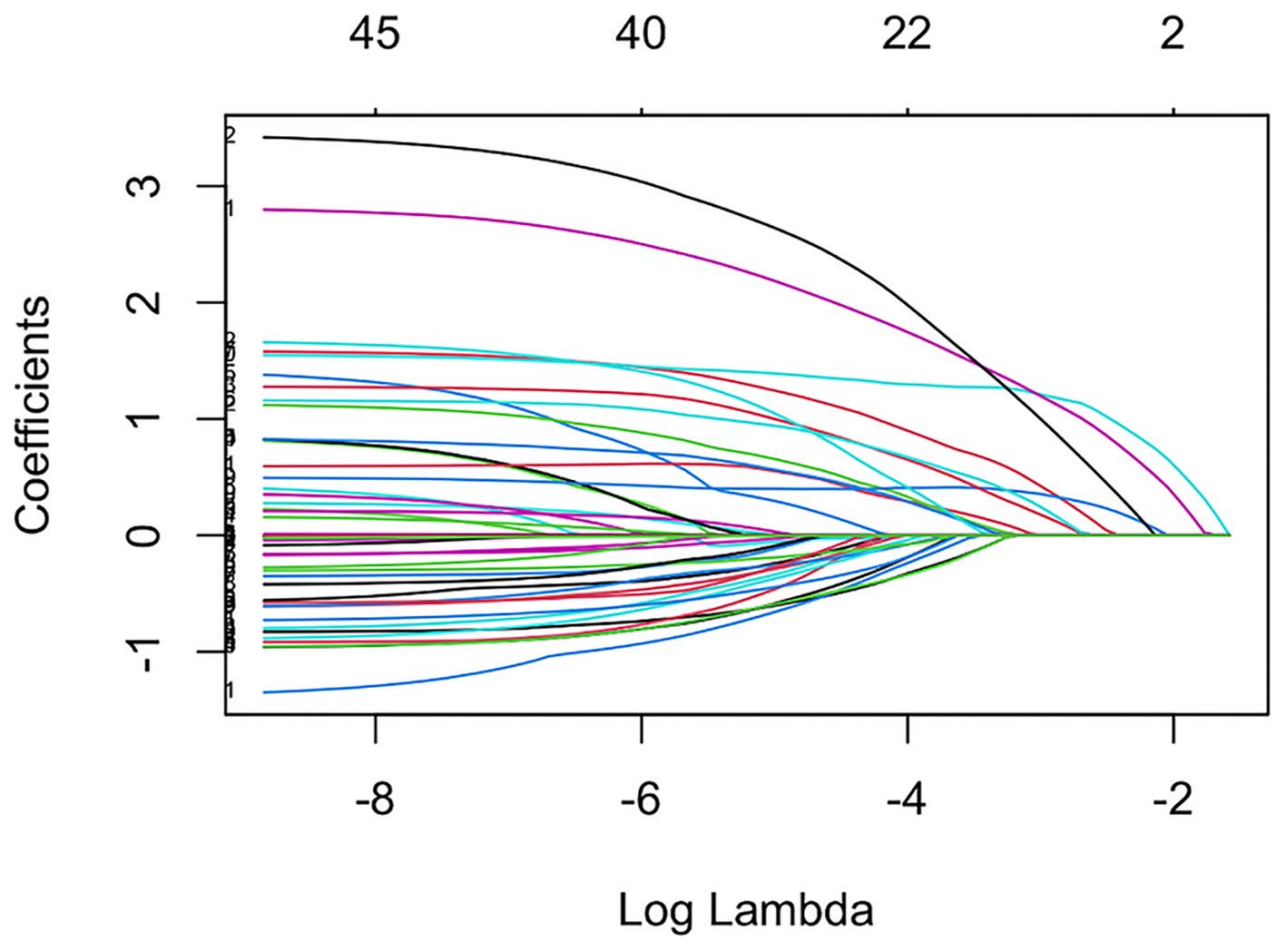
Regression coefficient vs. logarithmic lambda curve.

**Figure 2:**
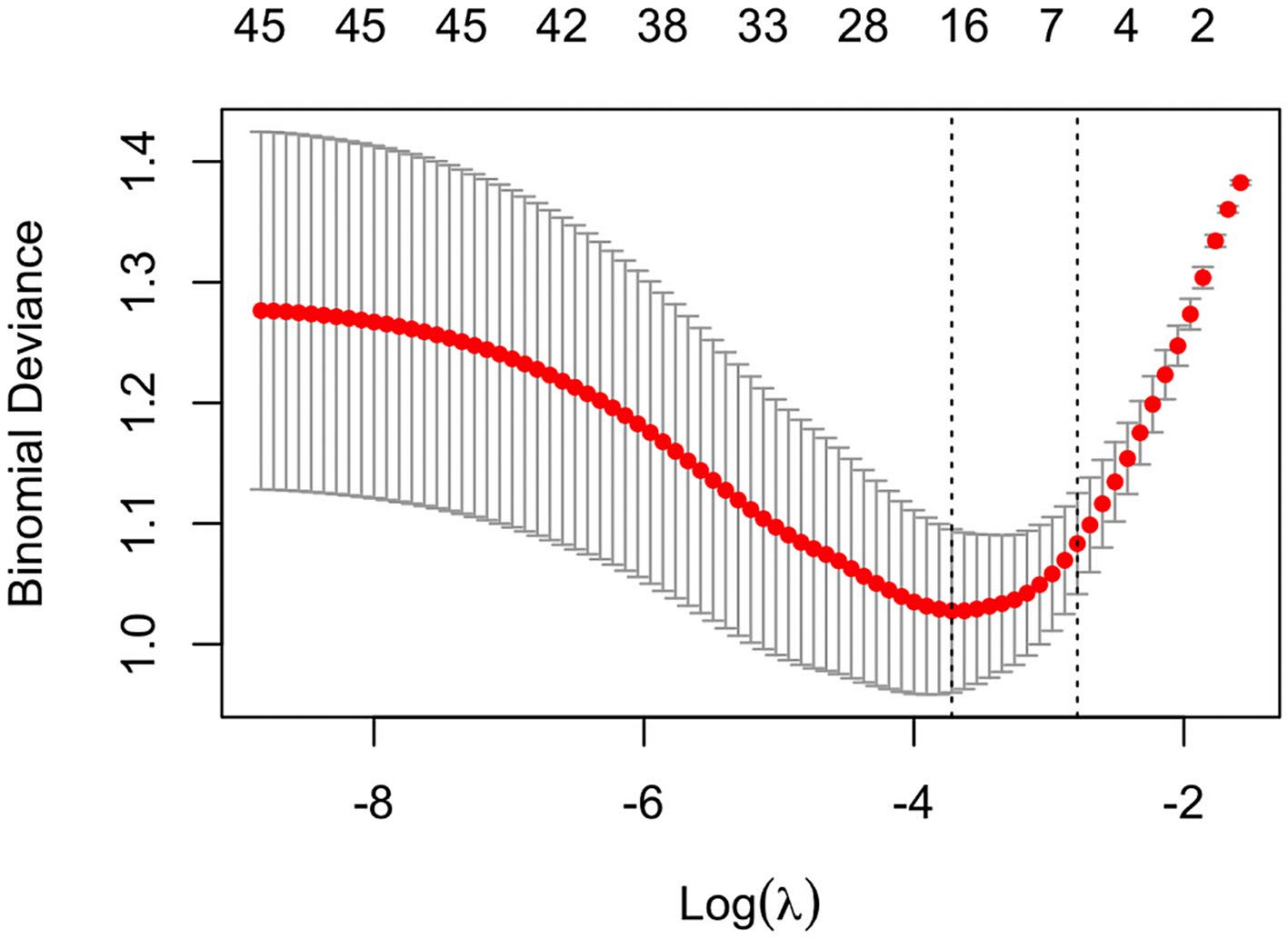
Lasso regression results plot.

All study participants were randomly divided into a training set (*n* = 315) and a test set (*n* = 135) using a stratified 7:3 sampling approach. In the training cohort, CIM presence served as the dependent variable, and feature selection was performed using LASSO regression. Ten-fold cross-validation was used to identify the optimal regularization parameter (*λ*), evaluating two key thresholds: λ.min, corresponding to the maximum AUC for the best model fit, and λ.1se, yielding a more parsimonious model with acceptable performance (Figure 1). The model, retaining 19 non-zero coefficients, maximized the AUC, balancing model complexity and generalizability (Figure 2). Nineteen variables were identified as significant predictors of CIM risk, including education level, smoking history, coronary artery disease, surgical history, metastasis (liver, bone, lymph nodes), radiotherapy history, chemotherapy cycles, nutritional status, and several laboratory indicators (WBC, Hb, PLT, NEUT, Lym, ALB, GLB, CA125, UA).

### 3.3 Model development and performance comparison

The 19 risk factors identified by LASSO regression were separately input into three machine learning algorithms—LR, RF, and XGB—to construct predictive models for CIM in CRC patients.

#### 3.3.1 Training set performance (*n* = 315)

All three models demonstrated good predictive performance in the training dataset. Both the LR and RF models achieved the highest accuracy of 0.76 (95% CI: 0.72–0.81). The LR model showed the best performance in terms of the AUC, reaching 0.84 (95% CI: 0.80–0.88), while the RF model had the highest F1 score of 0.76 (95% CI: 0.70–0.80), outperforming the other models.

**Figure 3:**
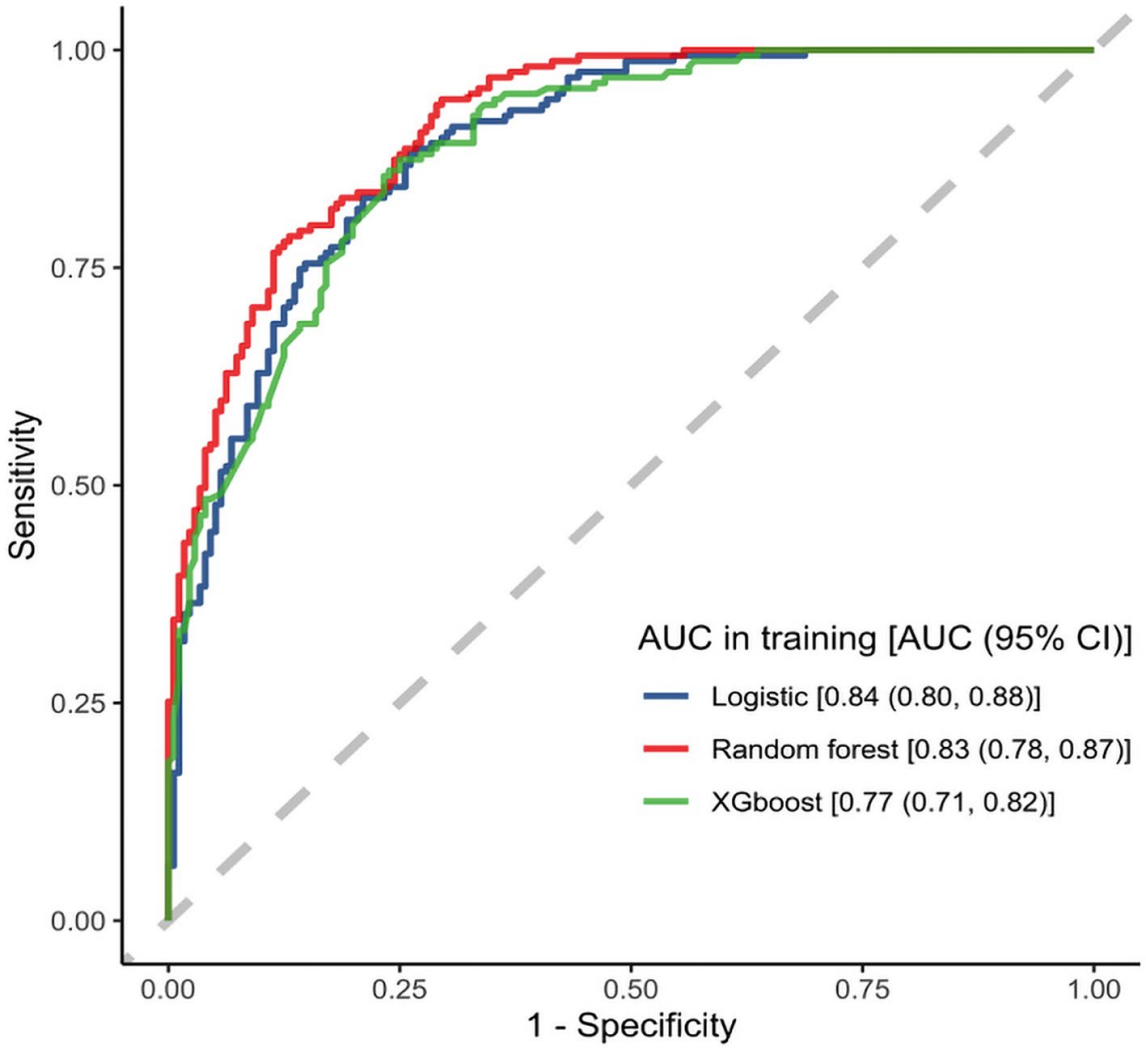
ROC plot of the training set.

Regarding sensitivity, LR and RF performed comparably, with values of 0.77 (95% CI: 0.71–0.84) and 0.77 (95% CI: 0.71–0.83), respectively. XGB exhibited the best specificity, reaching 0.77 (95% CI: 0.71–0.84). Detailed performance metrics for the three models are presented in Table 2, and their corresponding ROC curves are shown in Figure 3.

**Table 2:** Comparison of prediction performance evaluation indicators of different model training sets.

Model	Accuracy	AUC	F1-score	Sensitivity	Specificity
LR	0.76 (0.72, 0.81)	0.84 (0.80, 0.88)	0.75 (0.70, 0.81)	0.77 (0.71, 0.84)	0.75 (0.69, 0.81)
RF	0.76 (0.72, 0.81)	0.83 (0.78, 0.87)	0.76 (0.70, 0.80)	0.77 (0.71, 0.83)	0.76 (0.69, 0.81)
XGboost	0.75 (0.70, 0.80)	0.77 (0.71, 0.82)	0.74 (0.67, 0.79)	0.73 (0.66, 0.80)	0.77 (0.71, 0.84)

LR, logistic regression; RF, random forest; XGB, extreme gradient boosting; AUC, area under the receiver operating characteristic curve; F1, F1 score.

#### 3.3.2 Testing set performance (*n* = 135)

**Figure 4:**
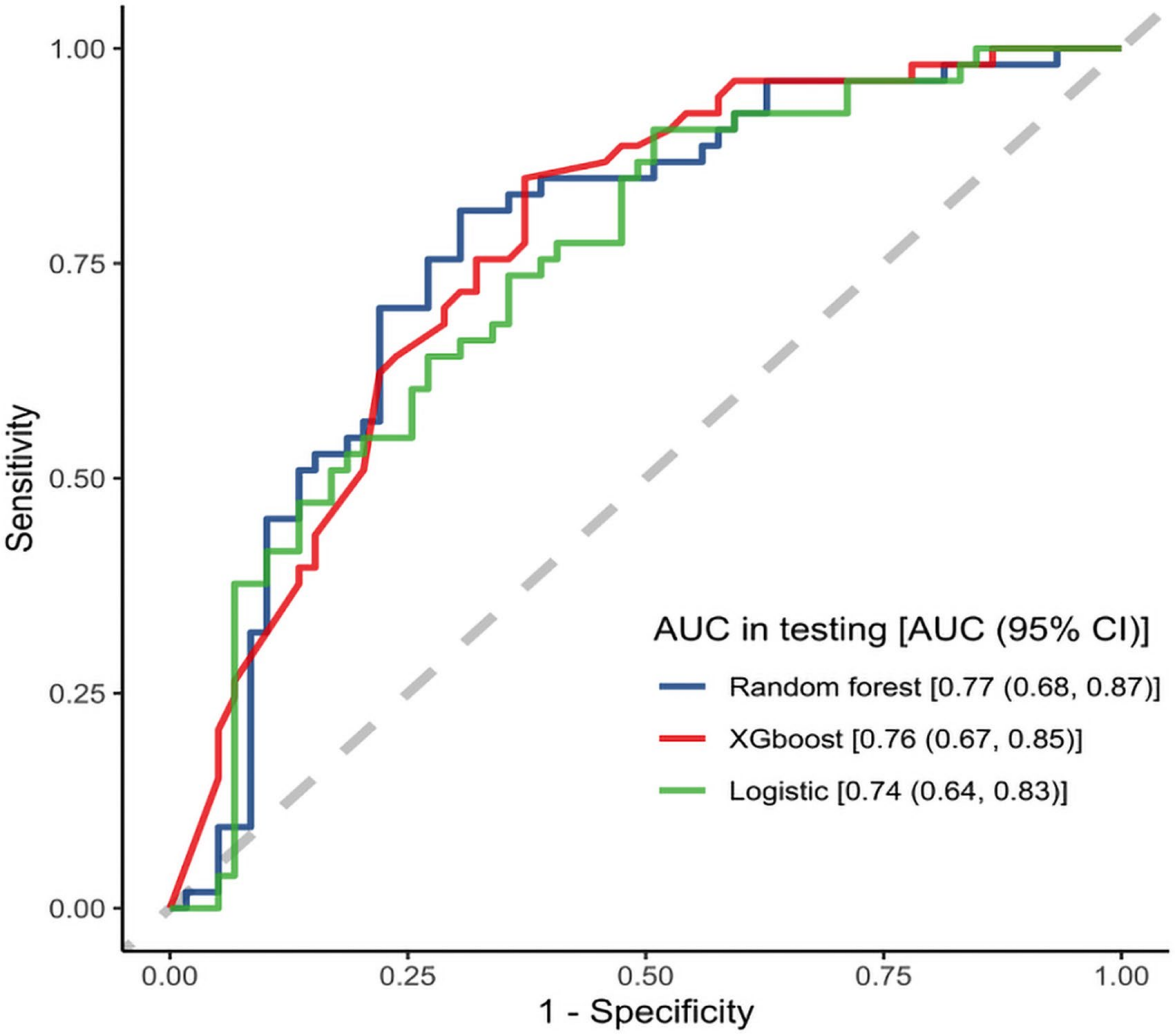
ROC plot of the test set.

In the testing dataset, all models demonstrated moderate predictive ability. Among them, the RF model outperformed others across multiple metrics: it achieved the highest accuracy of 0.71 (95% CI: 0.63–0.79), an AUC of 0.77 (95% CI: 0.68–0.87), an F1 score of 0.70 (95% CI: 0.60–0.79), and the best specificity of 0.73 (95% CI: 0.61–0.84). Sensitivity was comparable between the RF and XGB models, both reaching 0.70 (RF: 95% CI: 0.57–0.82; XGBoost: 95% CI: 0.58–0.83). Detailed test set results are summarized in Table 3, with corresponding ROC curves illustrated in Figure 4. Based on comprehensive performance in the test set, the RF model was selected as the optimal predictive tool.

**Table 3:** Comparison of prediction performance evaluation indicators of different model testing sets.

Model	Accuracy	AUC	F1-score	Sensitivity	Specificity
LR	0.66 (0.57, 0.75)	0.74 (0.64, 0.83)	0.65 (0.55, 0.75)	0.68 (0.55, 0.80)	0.64 (0.52, 0.76)
RF	0.71 (0.63, 0.79)	0.77 (0.68, 0.87)	0.70 (0.60, 0.79)	0.70 (0.57, 0.82)	0.73 (0.61, 0.84)
XGboost	0.70 (0.62, 0.78)	0.76 (0.67, 0.85)	0.68 (0.58, 0.78)	0.70 (0.58, 0.83)	0.69 (0.57, 0.81)

LR, logistic regression; RF, random forest; XGB, extreme gradient boosting; AUC, area under the receiver operating characteristic curve; F1, F1 score.

#### 3.3.3 Calibration analysis

**Figure 5:**
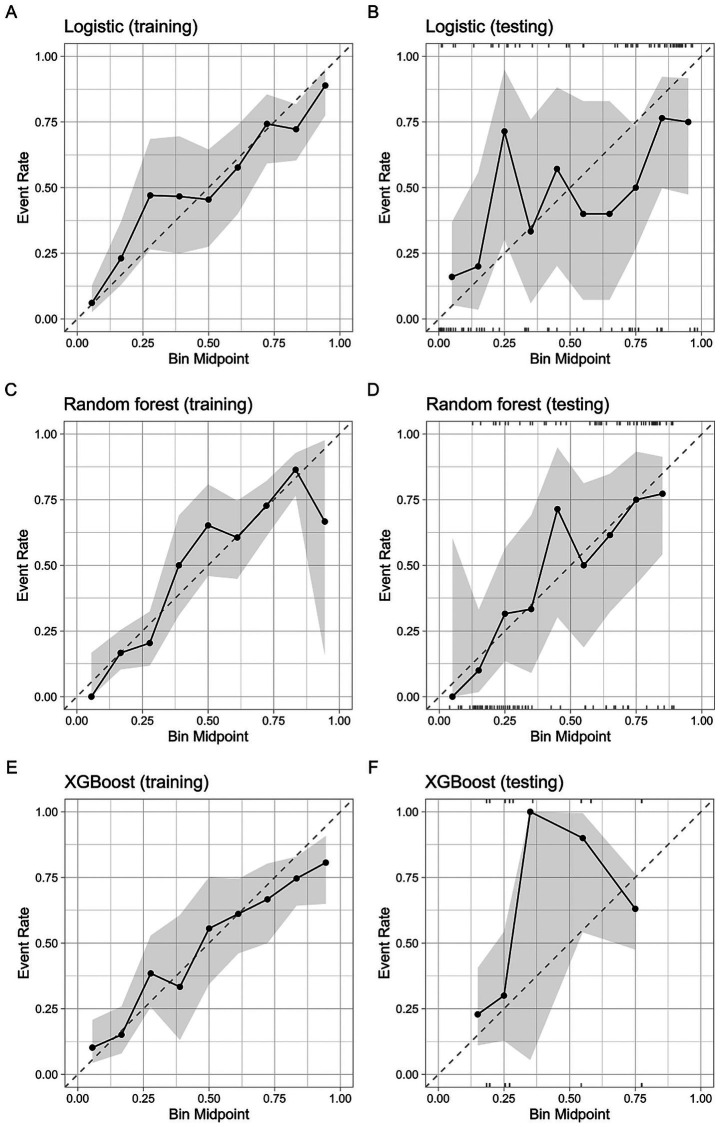
Calibration plots comparing the observed event rate against the predictive probabilities for the three machine learning models. **(A)** Logistic Regression model in the training cohort; **(B)** Logistic Regression model in the testing cohort; **(C)** Random Forest model in the training cohort; **(D)** Random Forest model in the testing cohort; **(E)** XGBoost model in the training cohort; **(F)** XGBoost model in the testing cohort. The dashed diagonal line represents perfect calibration (y=x), where the predicted probability equals the observed incidence. The black line with dots indicates the actual performance, and the gray area represents the 95% confidence interval.

Calibration curve analysis showed that all three models had good calibration in the training set, with LR and RF showing relatively better fit. However, their performance was less optimal in the test set. Detailed calibration plots for each model are provided in Figure 5.

### 3.4 External validation of the model

#### 3.4.1 Baseline characteristics of external validation cohort

A total of 150 CRC patients who received chemotherapy were included in the external validation cohort, comprising 98 patients from Feidong County People’s Hospital and 52 patients from Changfeng County People’s Hospital. The mean age of the entire cohort was 66.27 ± 11.83 years. Among them, 86 patients (57.33%) developed CIM, with a mean age of 67.56 ± 10.82 years; this group comprised 51 males (59.30%) and 35 females (40.70%). The remaining 64 patients (42.66%) did not develop CIM, with a mean age of 64.55 ± 12.96 years; 44 were males (68.75%) and 20 were females (31.25%).

Comparative analysis of baseline characteristics between the two groups revealed statistically significant differences (*p* < 0.05) in the following variables: hypertension, tumor history, bone metastasis, history of radiotherapy, prior history of myelosuppression, Nutritional Risk Screening 2002 (NRS2002) score, white blood cell count (WBC), hemoglobin (Hb), platelet count (PLT), neutrophil count (NEUT), lymphocyte count (Lym), and albumin (ALB) levels. Other variables showed no significant intergroup differences (*p* > 0.05), as summarized in Table 4.

**Table 4:** Comparison of baseline data between the two groups in external cohort [*n* (%), *M* (P25, P75), χ^2^ ± s].

Characteristic	Overall *N* = 150^1^	Non-myelosuppression *N* = 64^1^	Myelosuppression *N* = 86^1^	*p*-value^2^
Gender				0.2
Male	95 (63%)	44 (69%)	51 (59%)	
Female	55 (37%)	20 (31%)	35 (41%)	
Age	66.3 (11.8)	64.5 (13.0)	67.6 (10.8)	0.12
BMI	22.6 (3.3)	22.6 (3.4)	22.5 (3.2)	0.8
Educational level				0.061
Primary school or less	116 (77%)	44 (69%)	72 (84%)	
Junior high school	17 (11%)	8 (13%)	9 (10%)	
Senior high school	11 (7.3%)	7 (11%)	4 (4.7%)	
Junior college or above	6 (4.0%)	5 (7.8%)	1 (1.2%)	
Use of alcohol	22 (15%)	12 (19%)	10 (12%)	0.2
Use of tobacco	24 (16%)	12 (19%)	12 (14%)	0.4
Hypertension	49 (33%)	27 (42%)	22 (26%)	0.032
Diabetes	19 (13%)	8 (13%)	11 (13%)	>0.9
CHD	17 (11%)	8 (13%)	9 (10%)	0.7
With other tumors	16 (11%)	3 (4.7%)	13 (15%)	0.041
Prior operations	81 (54%)	36 (56%)	45 (52%)	0.6
Surgery method				>0.9
No surgery	39 (26%)	17 (27%)	22 (26%)	
Radical operation	94 (63%)	39 (61%)	55 (64%)	
Palliative operation	17 (11%)	8 (13%)	9 (10%)	
Stage of colorectal cancer				0.5
II stage	37 (25%)	18 (28%)	19 (22%)	
III stage	39 (26%)	14 (22%)	25 (29%)	
IV stage	74 (49%)	32 (50%)	42 (49%)	
Hepatic metastases	50 (33%)	24 (38%)	26 (30%)	0.4
Osseous metastasis	11 (7.3%)	1 (1.6%)	10 (12%)	0.025
Lymph node metastasis	106 (71%)	42 (66%)	64 (74%)	0.2
Metastatic tumor of lung	21 (14%)	11 (17%)	10 (12%)	0.3
Treatment regimen				0.5
Chemotherapy	99 (66%)	40 (63%)	59 (69%)	
Chemotherapy targeted therapy	47 (31%)	23 (36%)	24 (28%)	
Chemotherapy Immunotherapy	4 (2.7%)	1 (1.6%)	3 (3.5%)	
History of radiotherapy	23 (15%)	4 (6.3%)	19 (22%)	0.008
Chemotherapy cycle				0.3
Less than 6 M	66 (44%)	25 (39%)	41 (48%)	
More than 6 M	84 (56%)	39 (61%)	45 (52%)	
NRS2002				0.015
Less than 3	91 (61%)	46 (72%)	45 (52%)	
More than 3	59 (39%)	18 (28%)	41 (48%)	
WBC low	30 (20%)	3 (4.7%)	27 (31%)	<0.001
HB low	42 (28%)	2 (3.1%)	40 (47%)	<0.001
PLT low	24 (16%)	0 (0%)	24 (28%)	<0.001
NEUT low	32 (21%)	1 (1.6%)	31 (36%)	<0.001
Mono high	17 (11%)	10 (16%)	7 (8.1%)	0.2
Lym low	29 (19%)	6 (9.4%)	23 (27%)	0.008
ALB low	40 (27%)	8 (13%)	32 (37%)	<0.001
GLB high	3 (2.0%)	0 (0%)	3 (3.5%)	0.3
BUN low	7 (4.7%)	3 (4.7%)	4 (4.7%)	>0.9
CEA high	67 (45%)	34 (53%)	33 (38%)	0.072
CA199 high	46 (31%)	20 (31%)	26 (30%)	0.9
AFP high	3 (2.0%)	2 (3.1%)	1 (1.2%)	0.6
CA125 high	21 (14%)	6 (9.4%)	15 (17%)	0.2
TBIL	12.8 (11.1, 18.1)	12.8 (11.5, 18.8)	12.8 (10.7, 17.9)	0.3
Scr	64.5 (55.3, 79.7)	66.5 (55.3, 84.1)	63.5 (54.1, 74.0)	0.8
UA	300.0 (247.0, 370.1)	296.9 (247.0, 385.0)	300.5 (252.0, 365.0)	>0.9
GFR	102.0 (31.7)	106.5 (32.9)	98.7 (30.6)	0.9

1 Mean (SD), Median (Q1, Q3); *n* (%).

2 Pearson’s Chi-squared test; Wilcoxon rank sum test; Fisher’s exact test.

BMI, body mass index; CHD, coronary heart disease; NRS2002, Nutritional Risk Screening 2002; WBC, white blood cell; HB, hemoglobin; PLT, platelet; NEUT, neutrophil; MONO, monocyte; LYM, lymphocyte; ALB, albumin; GLB, globulin; BUN, blood urea nitrogen; CEA, carcinoembryonic antigen; CA199, carbohydrate antigen 19–9; AFP, alpha-fetoprotein; CA125, carbohydrate antigen 125; TBIL, total bilirubin; Scr, serum creatinine; UA, uric acid; GFR, glomerular filtration rate.

#### 3.4.2 Performance evaluation in external validation cohort

**Figure 6:**
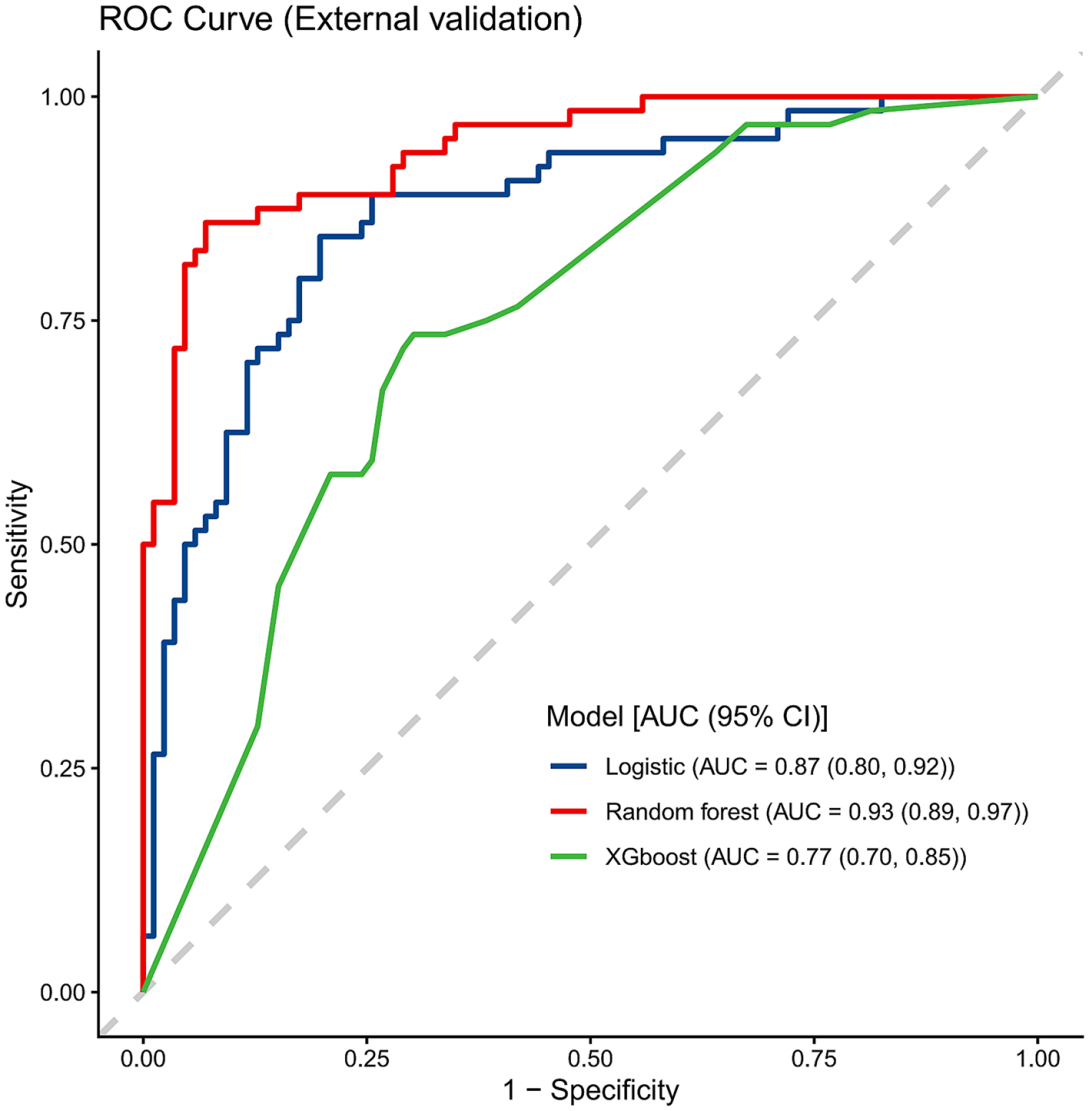
External validation ROC curve.

The three machine learning-based models developed for predicting CIM risk in CRC patients—LR, RF, and XGB—were evaluated on the external validation cohort. Among them, the RF model demonstrated superior performance. In the external dataset, the RF model achieved an AUC of 0.93 (95% CI: 0.89–0.97), reflecting high discriminative power (Figure 6). Furthermore, the model showed an accuracy of 0.89 (95% CI: 0.84–0.94), a sensitivity of 0.86 (95% CI: 0.77–0.92), a specificity of 0.92 (95% CI: 0.83–0.97), and an F1 score of 0.87 (95% CI: 0.80–0.93). However, the calibration analysis for the external cohort (Supplementary Figure S1) revealed noticeable fluctuations and relatively wide confidence intervals, likely stemming from the modest sample size of the external validation dataset.

### 3.5 Model interpretation

**Figure 7:**
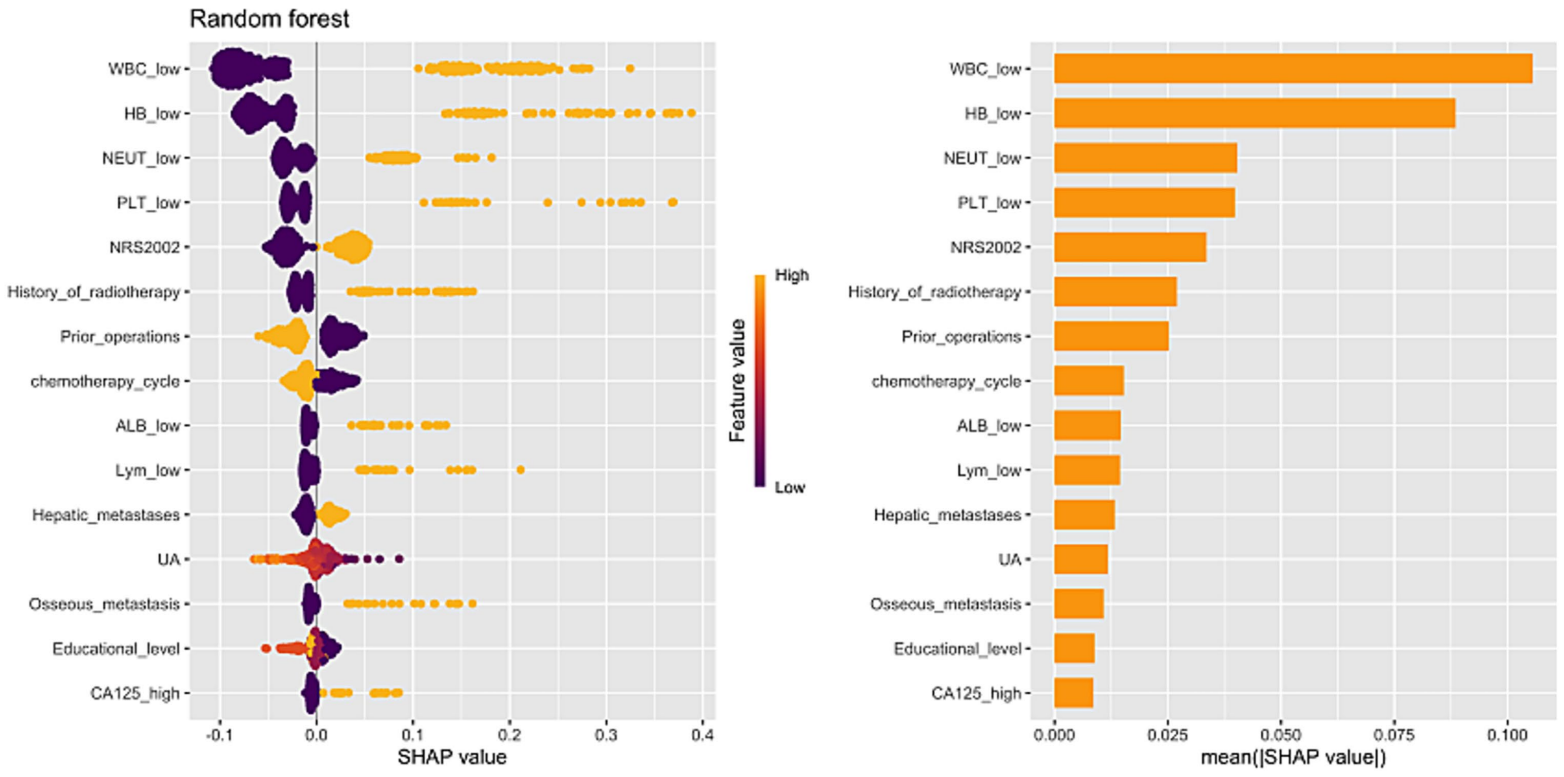
Random forest model SHAP algorithm important feature ranking.

To enhance interpretability of the CIM prediction model for CRC patients, we applied the SHAP algorithm to the best performing RF model, systematically evaluating the contribution of each predictor. As shown in Figure 7, WBC was the most influential predictor, followed by Hb, NEUT, PLT, NRS2002, and history of radiotherapy and surgery. A SHAP beeswarm plot was used to visualize the direction and magnitude of each variable’s effect, with the x-axis indicating effect size and the color gradient representing the variable’s actual value. Results showed that lower values of WBC, Hb, NEUT, and PLT were strongly associated with increased CIM risk, while higher NRS2002 scores and a history of radiotherapy, bone metastases, liver metastases, and low serum ALB levels were also significant risk factors.

## 4 Discussion

Our study revealed an overall incidence of CIM of 52.5% (236/450) among CRC patients receiving chemotherapy. This study developed and validated a machine learning-based prediction model for CIM in CRC patients, focusing on enhancing the accuracy of risk prediction through advanced algorithms. Our results demonstrate that the RF model outperformed both LR and XGB in terms of model performance. The RF model achieved an AUC of 0.93 in the external validation cohort. While this mathematically suggests strong discrimination, the relatively small size of this dataset warrants careful interpretation. Overall, these findings highlight the potential of machine learning in clinical oncology nursing, offering a significant improvement over traditional risk models and facilitating more informed, individualized patient care and proactive risk management.

In the performance comparison, the RF model consistently showed superior accuracy, sensitivity, and specificity compared to both LR and XGB. While LR demonstrated a relatively high AUC in the training cohort, it exhibited lower sensitivity, which is crucial in predicting high-risk patients who may suffer severe adverse effects, such as infections and treatment delays. Furthermore, while the RF model demonstrated strong calibration internally, its calibration curve in the external cohort exhibited significant fluctuations due to the limited sample size. The model’s ability to handle non-linear relationships between clinical features and outcomes without requiring predefined assumptions contributes to its robustness. These findings align with prior literature where RF has demonstrated significant advantages over other machine learning techniques (28, 29), particularly in handling complex, multi-dimensional clinical data.

The strength of this study lies not only in the predictive performance of the model but also in its interpretability. Through SHAP analysis, we provided transparency regarding the contribution of each variable in predicting CIM risk. SHAP analysis allowed us to identify key features that significantly influence the model’s predictions, offering insights into the underlying mechanisms of CIM development. WBC, Hb, NEUT, and PLT were identified as the most influential variables, which is consistent with existing literature that emphasizes the role of hematological parameters in myelosuppression risk (6). Analysis of feature importance shows that these baseline hematological indices are stronger predictors of CIM compared to tumor-related characteristics, reflecting the critical role of pre-treatment hematologic reserve. Additionally, the NRS2002 score, history of radiotherapy, and metastatic disease (particularly liver and bone metastasis) were also recognized as major risk factors. Although recorded only as a binary variable, radiotherapy in this colorectal cancer cohort almost exclusively represents pelvic radiation, which explains its strong impact on bone marrow and high predictive value. These results are consistent with prior studies (11, 12) that have linked poor nutritional status and cancer-related complications, such as metastasis and prior treatments, to increased toxicity during chemotherapy. By quantifying the direction and magnitude of each feature’s effect on CIM risk, SHAP analysis offers valuable, actionable insights for clinicians, making the model more interpretable and clinically relevant.

The clinical implications of these findings are substantial for proactive patient management. Machine learning models, particularly RF models, can offer the healthcare team an efficient tool for early identification of high-risk patients, enabling personalized supportive care strategies. For example, high risk patients could be flagged for more frequent hematological monitoring, early nutritional counseling, and targeted patient education regarding infection and bleeding precautions. Notably, we deliberately included Grade I toxicity as a critical early warning signal. While mild and rarely requiring medication, predicting it allows for proactive monitoring to prevent severe, life-threatening myelosuppression. Similarly, we assessed blood parameters on Day 7. Although the true neutrophil nadir for regimens like FOLFOX typically occurs later (Days 10–14), capturing the downward trend at Day 7 enables proactive intervention before the severe nadir occurs, rather than merely documenting the toxicity retrospectively.

However, this study has several limitations that should be considered. First, the model was developed and validated within a single geographic region; this regional clustering may limit its broad generalizability. Second, the retrospective design and lack of genetic (e.g., *DPYD*) and clinical predictors (e.g., performance status and dose intensity) may limit the model’s mechanistic depth. Notably, without accounting for dose intensity, the model might misattribute a lack of toxicity to physiological resilience rather than a clinically reduced drug dose. Finally, the external validation sample was determined by consecutive enrollment of eligible patients, resulting in a relatively modest size. This may lead to wider confidence intervals and impact the stability of the AUC values. Consequently, these metrics should be interpreted with caution as a preliminary evaluation, and our results primarily support early risk stratification rather than definitive guidance for high-grade toxicities.

Clinical implementation in primary care faces several barriers. First, limited IT infrastructure can be bypassed by deploying the model as a lightweight, web-based calculator rather than a complex system integration. Second, to overcome the ‘black-box’ nature of machine learning and build clinician trust, we utilized SHAP values to visualize feature contributions. Finally, data security concerns can be addressed by using local computation or encrypted platforms. Navigating these practical challenges is essential for transitioning the model into routine clinical workflows.

Future research should focus on several key areas. First, prospective validation in larger, multi-institutional cohorts is essential to confirm the model’s robustness before clinical adoption. Second, incorporating additional factors, such as genetic data (*DPYD*), tumor sidedness, and treatment history could further enhance predictive power and mechanistic depth. Finally, integrating this model into electronic health record (EHR) systems for real-time decision support remains a priority. Such advancements will facilitate the practical application of predictive tools in routine oncology care.

## 5 Conclusion

In this study, we developed CIM predictive models for CRC patients using LR, RF, and XGB algorithms, among which the RF model demonstrated the best overall performance. These findings suggest that the RF-based model can serve as a robust clinical decision-support tool. Deploying this model via web-based calculators or integrated electronic health record modules can provide real-time support, helping clinicians optimize monitoring and intervention strategies in primary care settings.

## Data Availability

The original contributions presented in the study are included in the article/Supplementary material, further inquiries can be directed to the corresponding author.

## Supplementary material

SUPPLEMENTARY FIGURE S1 Calibration curve of the RF model in the external validation cohort.
