# Supplementary figures and images for: Development and validation of a machine learning-based predictive model for chemotherapy-induced myelosuppression in colorectal cancer patients
Source: Front Med (Lausanne). 2026 Mar 25;13:1778951. doi: 10.3389/fmed.2026.1778951 (PMC13057565; doi:10.3389/fmed.2026.1778951)

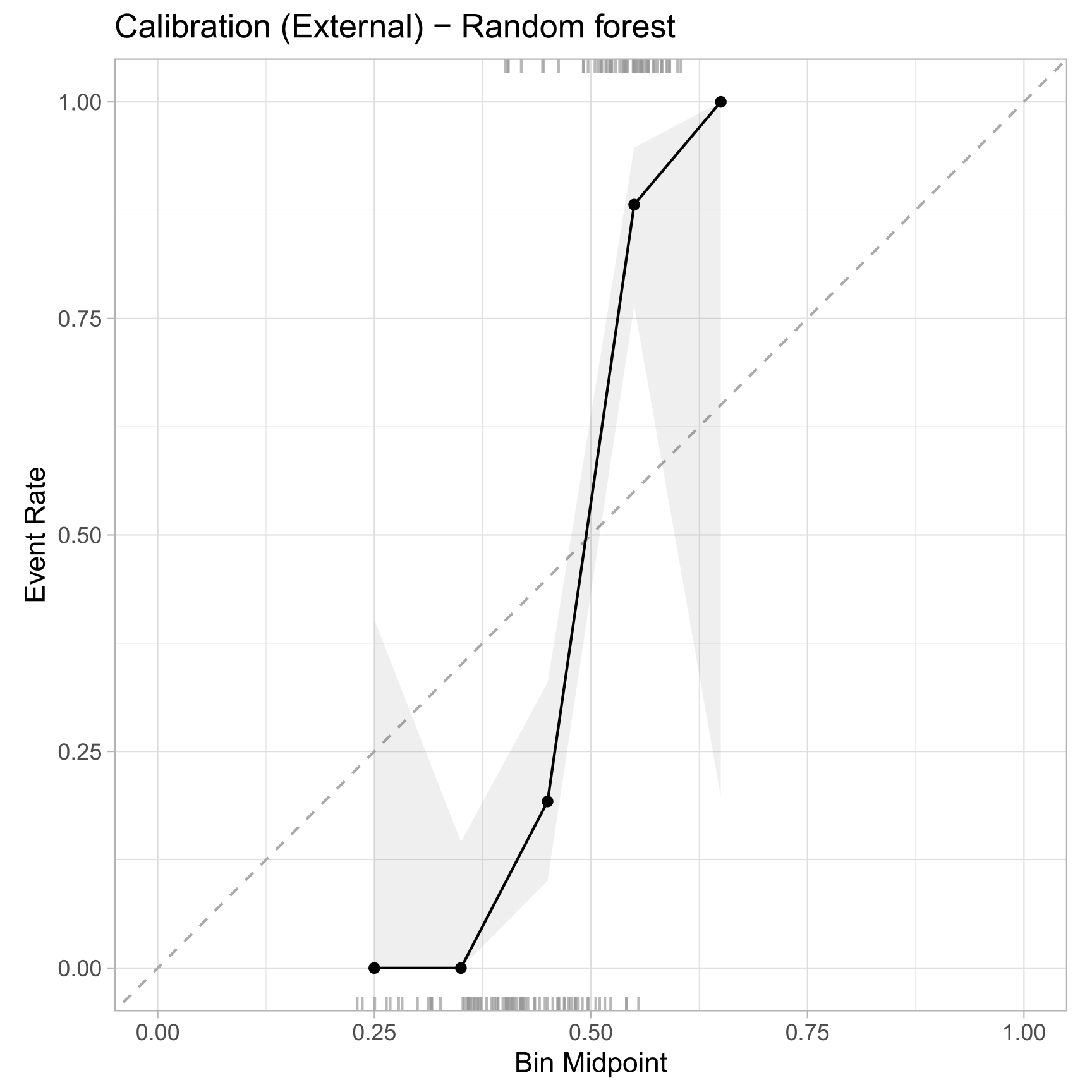

Supplement: SUPPLEMENTARY FIGURE S1 — Calibration curve of the RF model in the external validation cohort. [file image_1.tiff]
